# MiRNA Expression May Account for Chronic but Not for Acute Regulation of mRNA Expression in Human Thyroid Tumor Models

**DOI:** 10.1371/journal.pone.0111581

**Published:** 2014-11-06

**Authors:** Sébastien L. Floor, Aline Hebrant, Jaime M. Pita, Manuel Saiselet, Christophe Trésallet, Frederick Libert, Guy Andry, Jacques E. Dumont, Wilma C. van Staveren, Carine Maenhaut

**Affiliations:** 1 Institute of Interdisciplinary Research (IRIBHM), Free University of Brussels (ULB), Brussels, Belgium; 2 Welbio, Free University of Brussels, Brussels, Belgium; 3 Pitié-Salpêtrière Hospital, Université Pierre et Marie Curie, Paris, France; 4 Institut J. Bordet, Brussels, Belgium; University Hospital of Modena and Reggio Emilia, Italy

## Abstract

**Background:**

For thyroid tumorigenesis, two main human *in vitro* models are available: primary cultures of human thyrocytes treated with TSH or EGF/serum as models for autonomous adenomas (AA) or papillary thyroid carcinomas (PTC) respectively, and human thyroid tumor derived cell lines. Previous works of our group have assessed properties of those models, with a special emphasis on mRNA regulations. It is often assumed that miRNA may be one of the primary events inducing these mRNA regulations.

**Methods:**

The purpose of this study was to investigate the representativity of those models to study microRNA regulations and their relation with mRNA expression. To achieve this aim, the miRNA expressions profiles of primary cultures treated with TSH or EGF/serum and of 6 thyroid cancer cell lines were compared to the expression profiles of 35 tumor tissues obtained by microarrays.

**Results:**

Our data on primary cultures have shown that the TSH or EGF/serum treatment did not greatly modify the microRNA expression profiles, which is contrary to what is observed for mRNA expression profiles, although they still evolved differently according to the treatment. The analysis of miRNA and mRNA expressions profiles in the cell lines has shown that they have evolved into a common, dedifferentiated phenotype, closer to ATC than to the tumors they are derived from.

**Conclusions:**

Long-terms TSH or EGF/serum treatments do not mimic AA or PTC respectively in terms of miRNA expression as they do for mRNA, suggesting that the regulations of mRNA expression induced by these physiological agents occur independently of miRNA. The general patterns of miRNA expression in the cell lines suggest that they represent a useful model for undifferentiated thyroid cancer. Mirna probably do not mediate the rapid changes in gene expression in rapid cell biology regulation.

## Introduction

The last few years have seen the emergence of new cell regulators for mRNA gene expression: the microRNAs (miRNA). This class of small non-coding RNAs act on the stability and the translation efficiency of their target mRNA [1]. 19 to 25 nucleotides long, they are encoded by the genome as pri-miRNA and canonically processed by two RNAse III enzymes (Drosha and Dicer). They are then loaded into a RNA-induced silencing complex (RISC) which drives the selection of targets mRNA containing corresponding antisense sequences [2]. The single stranded miRNA loaded into the complex bind to their target sequences, most often located in the 3′ untranslated region of the mRNA, usually by only partial base paired complementarity [3]. miRNAs can repress mRNA expression through two main linked scenarios: direct mRNA destabilization or destabilization secondary to translational repression. Thereby, each miRNA can influence the expression of up to several hundred genes. On the other hand each mRNA can be targeted by several miRNA [3]. This is therefore not a one to one control but a multidirectional, pluritargeted, control network. This fits well with the concept of miRNA as attenuators or fine tuners of the cells. The growing number of known deregulated miRNA in tumors suggests that they may play an important role as a novel class of oncogenes or tumor suppressor genes [4].

Thyroid neoplasia include two types of benign tumors: hyperfunctioning autonomous adenoma (AA) and cold follicular adenoma (FTA). The former is characterized by a constitutive activation of the cAMP pathway, independent of the regulation exerted by thyrotropin (TSH), the hormone controlling thyroid activity. This hyperactivation can result in hyperthyroidism [5]. The latter one is characterized by a decrease of thyrocytes functional activity, of iodine metabolism and of secretion of thyroid hormones. There are also three malignant tumor types: the differentiated follicular (FTC) and papillary (PTC) carcinomas, and the dedifferentiated and highly aggressive anaplastic carcinomas (ATC) [6]. A certain number of FTA may evolve into FTC [6] and differentiated carcinoma (PTC and FTC) may progress towards the completely dedifferentiated ATC.

To investigate thyroid tumorigenesis, two main human *in vitro* models are available: primary cultures of human thyrocytes and human thyroid tumor derived cell lines. Human healthy thyrocytes can be cultured and stimulated by TSH, resulting in the activation of the cAMP pathway, which is constitutively activated in AA, or by EGF/serum, stimulating the RAS/RAF/MAPK signaling pathway, constitutively activated in PTC [7]. Two previous works of our group have shown that long term stimulations with TSH or EGF/serum mimic AA or PTC respectively, at the mRNA expression level [8], [9]. Human thyroid cancer cell lines, deriving from an initial tumor, are the most used *in vitro* models for thyroid tumors. Cell lines originating from ATC (8505C), FTC (FTC-133 and WRO) and PTC (TPC-1, BCPAP and K1) were included in this study. Previous works from our group have shown that these cell lines have evolved into a common, dedifferentiated phenotype. They may represent useful models for the dedifferentiated ATC [10]–[12]. The validity of these models with regard to miRNA expression has never been studied.

To address this question, miRNA expression profiling was performed in primary cultures treated with TSH or EGF/serum and in 6 thyroid cancer cell lines, and compared to the expression profiles in 35 thyroid tumor tissues (7 AA, 8 PTC, 8 FTC and 11 ATC).

We have shown that, while miRNA expression may to some extent account for chronic effects of tumorigenesis on mRNA expression, they do not account for mRNA short-term regulations by physiologic agents.

## Materials and Methods

### Primary cultures of thyrocytes

Thyroid tissue was obtained from the Erasme Hospital (Brussels, Belgium) from five patients, aged between 29 and 58 years, undergoing surgery for solitary or multiple nodules (multinodular goiter and hypofunctioning nodules). Nodules were always removed for pathologic analyses and only separate, healthy tissues were used for the preparation of the cell cultures. Follicles were prepared and plated on Petri dishes as described previously [8], [13]. For TSH treated cells, cultures were stimulated with 0.3 mU/ml bovine TSH (Sigma St. Louis, MO, USA) during 1.5, 3, 16, 24, 48 and 72 h [8]. For EGF/serum treated cells, cultures were stimulated with 25 ng/ml EGF (Sigma), and 10% of fetal bovine serum for the same times as for the TSH treated cells [9]. For both types of cultures, non-treated controls were included at each time point. Protocols have been approved by the Ethics Committee Erasme hospital (Protocol P2008/108). Written informed consent was obtained from all participants involved in the study.

### Cell line cultures

Tumor cell lines, originally described to be obtained from six different patients, were derived from two FTC (FTC-133 and WRO), three PTC (B-CPAP, K1 and TPC-1), and one ATC (8505C). The FTC-133, WRO, B-CPAP, and 8505C cell lines were a gift from Dr. G. Brabant (Medizinische Hochschule, Hannover, Germany) [14]. TPC-1 cells were obtained from Dr. M. Mareel (University of Gent, Gent, Belgium) and K1 from Pr. D. Wynford-Thomas (University of Wales, College of Medicine, Cardiff, UK) [14]. The STR authentification of these cell lines were done previously [11]. All cell lines were cultured as monolayers in a humidified atmosphere (5% CO2) at 37°C according to the instructions of their providers. WRO, B-CPAP, TPC-1 and 8505C were cultured in RPMI 1640 (with L-glutamine) supplemented with 10% fetal bovine serum, 2% streptomycin, 2% penicillin and 1% amphotericin B. FTC-133 and K1 were cultured in Dulbecco's Modified Eagle Medium, Nutrient mixture F-12 (1∶1, by volume) supplemented with 10% fetal bovine serum, 2% streptomycin, 2% penicillin and 1% amphotericin B. All culture reagents were purchased from Gibco (Paisley, UK). Expressions in cell lines were compared to expressions of a pool of human thyrocytes cultured for 4 days in the absence of stimulant.

### Thyroid tissue samples

For the investigation of microRNA expression profiles in a panel of benign and malignant thyroid tumors, samples of non-tumor and tumor thyroid tissues were obtained from patients undergoing surgery for thyroid disease. The cohorts of AA (n = 7) and of ATC (n = 11) used are described in Floor et al (submitted) and in Hebrant et al (submitted) respectively. PTC were obtained from the Erasme Hospital (Brussels, Belgium) and from the J. Bordet Institute (Brussels, Belgium) (n = 8). FTC were obtained from La Pitié-Salpêtrière Hospital (Paris, France) (n = 8). Final diagnoses were made by pathologists. All tissues were immediately dissected, placed on ice, snap-frozen in liquid nitrogen, and stored at −80°C until processing. The ethics committees of the institutions approved the protocol. Expressions of pathological thyroid samples were compared to normal contralateral tissues or in the case of ATC to a pool of normal thyroid tissues. Protocols have been approved by the Ethics Committee Erasme hospital (Protocol P2008/108). Written informed consent was obtained from all participants involved in the study.

### RNA purification

Total RNA was extracted using a TRIzol Reagent kit (Invitrogen) followed by purification on miRNeasy columns (Qiagen, Hilden, Germany). The RNA concentrations were spectrophotometrically quantified, and their integrity was verified using an automated electrophoresis system (Experion, Bio-Rad).

### miRNA microarrays

1 µg of total RNA was engaged for the hybridization reaction. Briefly, total RNA was labeled using the miRCURY LNA microRNA Power Labeling Kit (Hy3/Hy5) (Exiqon, Copenhagen, Denmark), according to the manufacturer's protocol. Labeled RNA was purified on a miRNeasy column (Qiagen), and samples were hybridized using Corning Pronto! Microarray Hybridization Kit onto in-house-printed slides with the miRCURY LNA microRNA ready-to-spot probes set (V11.0 according to Mirbase 11.0, containing 841 human miRNA) from Exiqon. All hybridizations were performed in duplicates with dyes swapped. After overnight hybridization, microarray slides were washed under stringent conditions: twice 60 s at 60°C with 2X SSC and 2% SDS, twice 60 s at 60°C with 2X SSC and twice 60 s at room temperature with 0.2X SSC. The slides were then scanned using a GenePix 4000B scanner (Axon, Sunnyvale, CA).

### Bioinformatics and Statistical Analysis of Microarray Data

Expression levels were quantified with GenePix Pro 6.0 (Axon). Treatment of the raw data was carried out by using the LIMMA 3.4.5 package for the R 2.8.1 language [15]. A Normex + offset background correction and LOESS normalization were performed. Data points were removed when intensity values for both dyes were below 200% of the background and absent calls were removed before subsequent statistical analyses. The statistical analyses of the microarray data were performed using Significance Analysis of Microarray (SAM) [16]. All the spots with a fold change > |1.5|, with a *q*-value<5% and present in at least 80% of the samples were considered as regulated. All the representations are performed with R 2.8.1 or 2.14.1 language. The method used to compute the distance for the hierarchal clustering was “Ward”. The function used for the PCA is prcomp.

### Quantitative RT-PCR for NIS mRNA expression

NIS (sodium iodide symporter) mRNA expression was quantified in treated and non-treated primary cultures by qRT-PCR. After a DNase treatment using DNase I Amplification Grade (Invitrogen), reverse transcription was performed using Superscript II RNase H Reverse Transcriptase (Invitrogen) following the manufacturer's protocol. Oligonucleotide sequences corresponding to the NIS transcript were designed using Primer Express software (Applied Biosystems, Foster City, CA, USA). The forward sequence was: 5′-TGCTCTTCATGCCCGTCTTC-3′, and the reverse one: 5′-AGCGCATCTCCAGGTACTCGT-3′. The qRT-PCR products were run on an Applied Biosystems 7500 Fast Real Time PCR with SyberGreen (Applied Biosystems). NEDD8 and TTCI mRNA expression were used for normalization [17].

### Quantitative RT-PCR for miRNA

Validations of the regulation of 4 miRNA from microarray data were performed using TaqMan MicroRNA Assay kits according to the manufacturer's protocol (Applied Biosystems). miRNA-specific cDNA were generated from 10 ng of total RNA using the TaqMan microRNA RT Kit and the gene-specific RT primers from the TaqMan microRNA Assays (Applied Biosystems) according to the manufacturer's instructions. All reactions were run in triplicate. Relative quantification (RQ) was calculated based on the Pfaffl Method [18]. Ct data were normalized with an internal control, U6 snRNA.

## Results

### Contrary to previously described mRNA data, miRNA expression profiles are not greatly modified in primary cultures treated with TSH or EGF/serum

In order to investigate the miRNA expression profiles in primary cultures treated with TSH or with EGF/serum, human primary thyrocytes cultures were treated with 0.3 mU/ml TSH or with 25 ng/ml EGF and 10% serum for 1.5, 3, 16, 24, 48 and 72 h. TSH essentially activates the cAMP pathway and stimulates differentiation and proliferation of the thyrocytes [8], [19]. The EGF/serum treatment essentially activates the MAPK pathway and has a stimulatory effect on proliferation but an inhibitory effect on differentiation [9], [20]. To ensure that the primary cultures has responded to the treatments, the level of NIS mRNA (a downstream element of the pathways modulated by TSH or EGF/serum treatment of thyrocytes) was quantified by qRT-PCR after 48 or 72 h of treatment (Figure S1). As expected, NIS mRNA was strongly up-regulated in response to TSH treatment compared to non-treated cells, and down-regulated in response to EGF/serum treatment [21].

Each treated sample was hybridized against its corresponding non-treated control at each time point. To compare these treated primary cultures with the tumor tissues, miRNA purified from 7 AA and 8 PTC were also hybridized against their respective adjacent normal tissues. miRNA expression data of treated primary cultures (two or three different cultures), and of the tumors were first filtered as described in Materials and Methods. A miRNA was then considered as regulated if the mean of its regulation was higher than 1.5 fold and a q-value <5%. For all the subsequent analyses, we have only considered the 397 miRNA that were consistently expressed in the primary cultures, and compared their expression in the tumor tissues.

As shown in Table 1, the different treatments performed did not greatly modify the miRNA expression profiles of the cultured cells (871 miRNA measured on the microarrays). For the TSH treatment, no miRNA was modulated before 48 hours, and 2 miRNA were downregulated (hsa-miR-492 and hsa-miR-584) at that time. Another miRNA (hsa-miR-138) was also downregulated after 72 hours of TSH treatment (Table1). None of those 3 miRNA was found as regulated among the AA studied. For the EGF/serum treatment, no miRNA was regulated before 24 hours of treatment. At that time, 2 miRNA were upregulated (hsa-miR-668 and hsa-miR-374b-3p), and one downregulated (hsa-miR-370). After 48 and 72 hours of treatment, hsa-miR-143 showed an upregulation in the treated cultures (Table 1). Again, the 4 miRNA whose expression was modulated upon EGF/serum treatment were not regulated in PTC. On the contrary, in our previous results 149 or 57 mRNA were deregulated after 24 and 48 hours of TSH or EGF/serum treatment respectively (2122 mRNA measured). The majority of them were similarly modulated in AA for the TSH treatment and in PTC for the EGF/serum treatment [8], [9].

**Table 1:** miRNA regulated in primary culture (qRT-PCR).

TSH 48 h	*miRNA*	*Log Ratio*
	hsa-miR-492	−0,73
	hsa-miR-584	−0,64
TSH 72 h	*miRNA*	*Log Ratio*
	hsa-miR-138	−0,62
EGF/serum 24 h	*miRNA*	*Log Ratio*
	hsa-miR-370	−0,66
	hsa-miR-668	0,65
	hsa-miR-374b*	0,83
EGF/serum 48 h	*miRNA*	*Log Ratio*
	hsa-miR-143	0,70
EGF/serum 72 h	*miRNA*	*Log Ratio*
	hsa-miR-143	0,69

**Figure 1:**
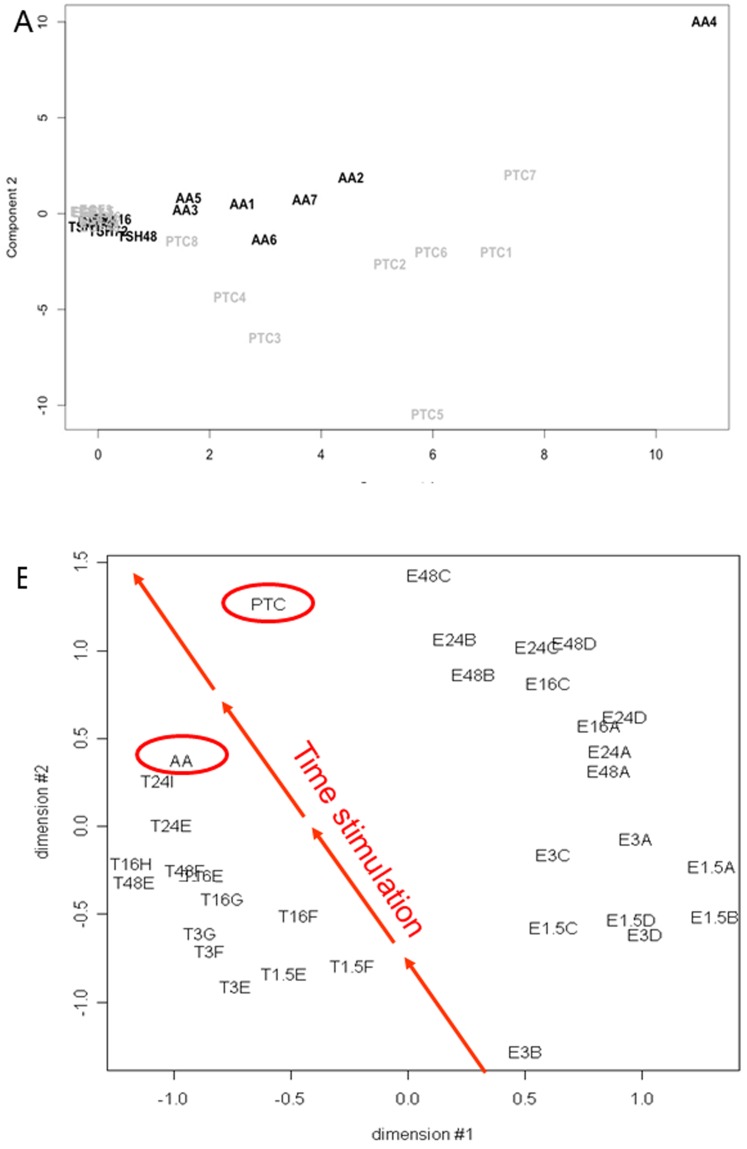
A: Principal Component Analysis of the miRNA expression data from primary cultures stimulated for 1,5, 3, 16, 24 and 48 hours with TSH or with EGF/serum and from tumor tissues (autonomous adenomas (AA) and papillary thyroid carcinomas (PTC)). The two main components are shown. All the treated primary cultured samples are localized close to each other near the origins of the axis, illustrating the low change on the miRNA expression resulting from the treatment. The proportion of variance explained by the Principal Component 1 and 2 are 0.353 and 0.270, respectively. The PCA was generated with R 2.8.1 with the function Prcomp. **B: Multi-dimensional scaling analysis (MDS) based on four independent human primary thyroid cell cultures stimulated with 25 ng/ml EGF and 10% serum (E) for 1.5, 3, 16, 24 and 48 hours and on five independent human primary thyroid cultures stimulated with 0.3 mU/ml TSH (T) for similar times [32]** **.** Expression profiles from a pool of autonomous adenomas (AA) and of a group of papillary thyroid carcinomas (PTC) were included. The MDS is based on all the spots present on the array. The distortion of distances (stress) between the MDS 2D space and the actual gene space is 19.6%.

The miRNA and mRNA expression profiles of the primary cultures treated with TSH or with EGF/serum and the tumor tissues can be represented on a PCA (Principal Component Analysis) (Figure 1A). The PCA was based exclusively on the miRNA that were expressed in treated primary cultures. This representation showed that all the primary cultures are close to each other and are located near the origin of the axis, reflecting the small differences in miRNA expression profiles between treated and untreated thyrocytes. Moreover PTC and AA were clearly separated, with the AA somewhat closer to the primary cultures than the PTC. This is to be compared to our previous data on mRNA expression which clearly showed a temporal evolution patterns of short-term to long-term TSH or EGF/serum stimulated cells to AA and PTC respectively (Figure 1B).

### miRNA of primary cultures treated with TSH evolved with the duration of the stimulation but such an evolution was not clear in EGF/serum treated cultures

**Figure 2:**
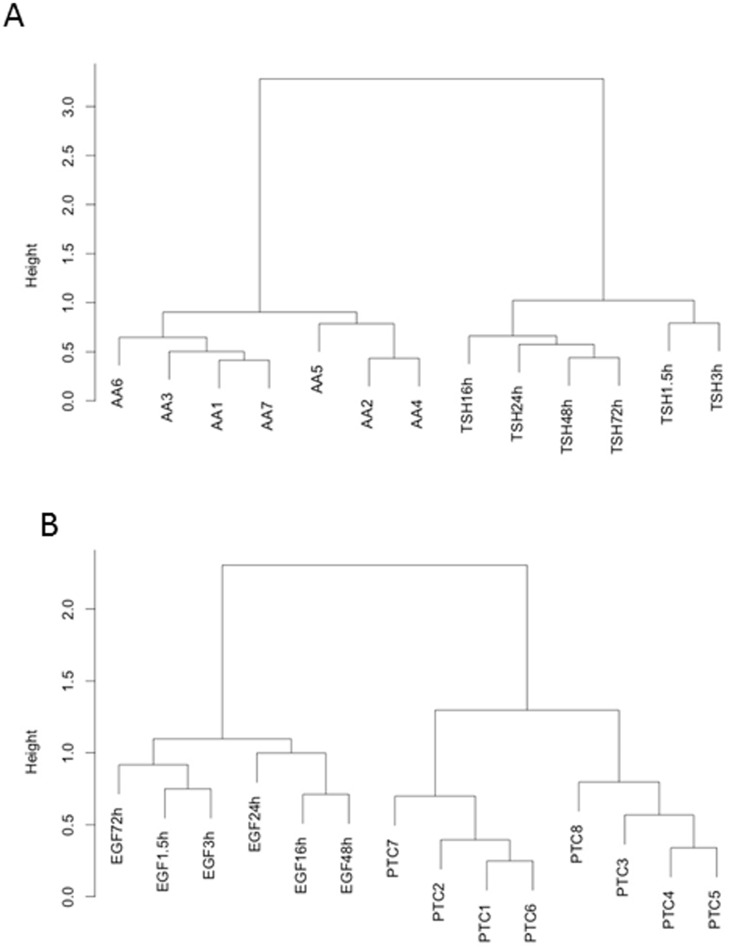
Hierarchical clustering on the miRNA expression data from primary cultures treated with TSH or with EGF/serum and from tumor tissues. Hierarchical clusterings were generated using R 2.8.1. The method used to compute the distance was “Ward”. A. Hierarchical clustering on the data from the primary cultured thyrocytes stimulated with 0.3 mU/ml of TSH for 1.5, 3, 16, 24, 48 and 72 hours and from 7 autonomous hyperfunctioning adenomas (AA). A time evolution in miRNA expression according to TSH treatment of primary cultured thyrocytes is observed suggesting fine changes in miRNA level. The AA cluster apart from the TSH treated primary cultures indicating a clear separation between those two groups of samples. Each time point is the mean of triplicates. B. Hierarchical clustering on the data from primary cultured thyrocytes stimulated with 25 ng/ml EGF and 10% serum for 1.5, 3, 16, 24, 48 and 72 hours and from 8 papillary thyroid carcinomas. In this case, no temporal evolution could be observed by hierarchical clustering. Each time point is the mean of triplicates. Tumors and treated primary cultures clustered apart, suggesting that primary cultures evolved differently according to the treatment but that there was no convergence to AA or PTC for the long-term treatment.

The miRNA expression profiles of the stimulated primary cultures were further analyzed using hierarchical clustering. The hierarchical clusterings were based on the miRNA expressed in the treated primary cultures. First, we studied the evolution of the miRNA expression profiles in the primary cultured thyrocytes stimulated with TSH. A clustering performed on the miRNA expression data from those samples shows firstly a separation between the short-term (1,5 and 3 h) and the long-term TSH treatment groups (16, 24, 48 and 72 h), and secondly a time course evolution in miRNA expression. This suggests that fine changes occur at the miRNA level in those cells (Figure 2A). Such an evolution is less clear in the case of EGF/serum treated cells (Figure 2B).

### Contrary to mRNA, miRNA patterns from long-term TSH or EGF/serum treatments of thyrocytes do not mimic those of AA or PTC respectively

When comparing, by hierarchical clustering analysis, the miRNA expression in TSH treated primary cultured thyrocytes and AA (Figure 2A), the tumors clustered apart from the treated cells. The size of the branches indicated that there is a clear separation between the two groups of samples, similar to what was observed in the PCA (Figure 1). This is contrary to what observed previously for mRNA expression [8] where long-term TSH stimulated cultures clustered together with the AA (Figure 1). Such cluster was not observed for miRNA.

For the EGF/serum treated thyrocytes (Figure 2B), no temporal evolution upon the treatments was observed by hierarchical clustering. Again, cultured thyrocytes and PTC were clearly separated: the pattern of late miRNA expression did not converge to the pattern of PTC, contrary to what was observed for the mRNA [9] (Figure 2B).

### Global miRNA and mRNA expression profiles of cell lines showed that they cluster together with ATC but not with differentiated tumors

The most commonly used *in vitro* model of thyroid tumors are thyroid tumor derived cell lines. 6 human thyroid tumor cell lines were investigated in this study: FTC-133 and WRO derived from FTC, B-CPAP, K1 and TPC-1 derived from PTC and 8505c derived from ATC. The thyroid origin of all these cell lines studied has been formally confirmed [14], [22]. The miRNA expression profiles of these cell lines were studied by microarray (with Exiqon library V11.0), and compared to a pool of non-treated primary cultures of thyrocytes. These expression profiles were then compared to the profiles of 8 FTC, 8 PTC and 11 ATC. Only the miRNA expressed in the cell lines were taken into account for the analyses.

**Figure 3:**
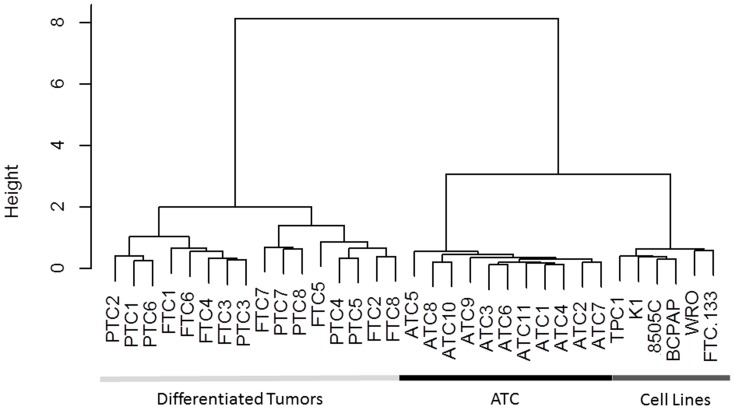
Hierarchical clustering of the microarray data from the 6 cell lines and from different malignant thyroid tumors (PTC, FTC and ATC). Hierarchical clustering was generated using R 2.14.1. The method used to compute the distance was “Ward”. All the cell lines clustered together, with the ATC showing that the cell lines are closest to the dedifferentiated tumors than the tumors from which they derived. The data from the six cell lines were the mean of triplicate experiments. Differentiated tumors refer to papillary (PTC) and follicular (FTC) thyroid carcinomas.

Hierarchical clustering showed a clear separation into 2 groups of samples (Figure 3). One branch of the cluster included all the differentiated tumors (PTC and FTC), whereas the other one contained the ATC and the six cell lines. This indicated that the cell lines are closer to each other than to the type of tumor they derived from. Their miRNA expression profiles showed a greater similarity with the dedifferentiated ATC than with the differentiated PTC and FTC. This is in line with the data obtained for the mRNA.

### 19 miRNA are commonly deregulated in all the investigated cell lines, and 18 of them are similarly deregulated in ATC

**Figure 4:**
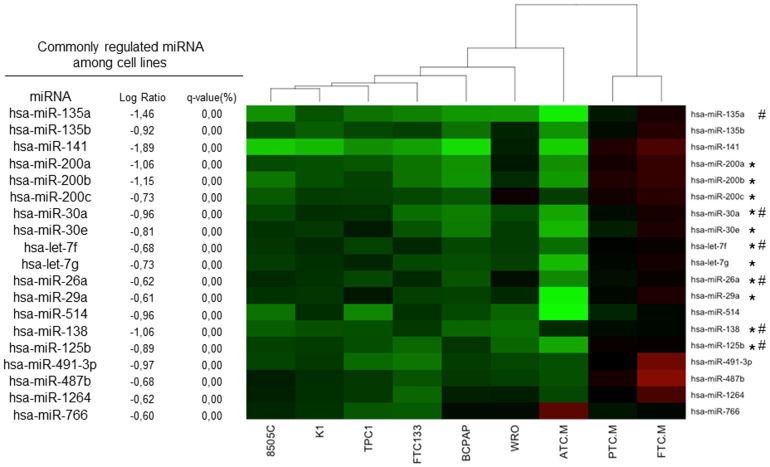
Heatmap based on the results of SAM one class analysis of the microarray data from 6 thyroid cancer cell lines and compared to tumor tissues. 18 of the 19 modulated miRNA in cell lines are modulated in the same way in ATC, but are not modulated or in an opposite way in differentiated tumors (PTC and FTC). The heatmap was generated using R 2.14.1. with the library “gplots”. ATC.M (n = 11), PTC.M (n = 8) and FTC.M (n = 8) refer to the mean of the miRNA data from the different tumors analyzed. * miRNA described as regulated in ATC by Braun et al [23]. # miRNA described as regulated in ATC by Visone et al. [24].

SAM (Significance Analysis of Microarray) one class was used to obtain a list of miRNA commonly modulated among the 6 cell lines. A miRNA was considered as commonly regulated if a fold change > |1.5| and a *q*-value <5% was observed in all the cell lines. Among the 6 cell lines, 19 miRNA were commonly downregulated (Figure 4). Among these modulated miRNA, some are part of the same miRNA families: miR-135a and miR-135b are members of the miR-135 family; miR-200a, miR-200b, miR-200c and miR-141 belong to the miR-200 family; miR-30a and miR-30e to the miR-30 family; and let-7f and let-7g to the let-7 family. No commonly upregulated miRNA was identified.

In order to validate the deregulations observed by microarray, qRT-PCR analyses were performed. The expression of 4 miRNA (3 down-regulated: hsa-miR-30a, hsa-miR-29a and hsa-miR-125b and one non-regulated: hsa-miR-659) was investigated in the 6 cell lines, compared to a pool of non-treated primary cultures (Figure S2). All the expression patterns obtained by qRT-PCR were concordant with the microarray data.

The expression of those 19 commonly regulated miRNA was then compared with tumor tissues. Apart from miR-766, all the other miRNA were also down-regulated in ATC (Figure 4). By contrast, none of them was down-regulated in PTC or FTC. 11 of the 19 miRNA commonly regulated in the cell lines are regulated in the same way in the 3 ATC analyzed by Braun et al. [23], and 6 miRNA were reported as modulated in the ATC by Visone et al. [24]. This extends at miRNA level our previous observation that the cell lines are closer to the ATC than to the tumors they are derived from [11].

## Discussion

Three major conclusions can be drawn from these results: 1) While long-term (24 h, 48 h) EGF/serum or TSH stimulated primary cultures of human thyrocytes are good *in vitro* models for PTC and AA respectively for mRNA expression, they are not for miRNA expression. 2) Human cancer cell lines are better models for ATC than for their tumor of origin. 3) miRNA probably do not mediate the rapid changes in gene expression.

*In vitro* models, such as primary cultured thyrocytes or cancer cell lines, are primordial for research, but a good characterization prior to their use as a model for the corresponding tumor is essential. *In vitro* models of thyroid tumors are well determined and different publications of our group have explored their validity, essentially by mRNA microarray analyses [8]–[11]. Although the cell lines are used routinely to investigate the role of mRNA or miRNA identified as modulated in tumors, until now no such systematic study concerning miRNA expression in those models has been performed.

First, primary cultures of human healthy thyrocytes treated with TSH or with EGF/serum were investigated. This represents one of the most physiological models that allow human thyroid tumorigenesis investigation. Previously our group has shown that relatively long (16, 24 and 48 h) TSH treatments of thyrocytes represent a good model to investigate benign AA [8]. Similar conclusions for the PTC model were obtained when the thyrocytes were treated with EGF/serum [9]. In this study, we have investigated if similar conclusions could be drawn when regarding the miRNA expression in these models. To answer this question, the same culture conditions used in the two previous studies have been reproduced and miRNA expression analysis has been performed by microarrays.

The first striking observation was the small number of miRNA regulated by TSH or EGF/serum in primary cultures, showing the weak impact of those treatments on miRNA expression profiles in such human thyroid cells. Considering a fold-change of 1.5, only three miRNA were downregulated following TSH addition (hsa-miR-492 and hsa-miR-584 at 48 h and has-miR-138 at 72 h) and no miRNA was upregulated. No overlap between the 3 TSH deregulated miRNA and the 12 miRNA downregulated in AA was observed. A similar conclusion could be drawn for EGF/serum treated thyrocytes, where there was no overlap with the PTC deregulated miRNA.

The weak impact of the treatments on miRNA expression was also confirmed by the PCA. Indeed, the primary cultures treated with TSH or EGF/serum were all close to each other, reflecting very little differences in miRNA profiles. In the two publications describing mRNA expression in these models [8], [9], the longest stimulation time was 48 h, whereas in the present study, we stimulated our cultures up to 72 and 96 h (data not shown), but still no further miRNA regulation was detected.

Although few regulated miRNA showed a fold change of 1.5 or higher, an effect on the global miRNA expression profiles could still be detected according to the time of stimulation with TSH (Figure 2). This effect may result from the addition of numerous small changes in miRNA expression. Thus a temporal evolution in miRNA expression could be observed following TSH treatment. However this was not the case for the EGF/serum treatment.

Thus our data indicate that, contrary to what was reported for mRNA [8], [9], long- terms TSH or EGF/serum treatment of primary cultured thyrocytes do not mimic AA or PTC, respectively, in term of miRNA expression. Our work contrasts with three recent publications [25]–[27] reporting that TSH induced mainly a decreased expression of miRNA implicated in cell proliferation in rat thyroid cell lines (PCCL3 and FRTL5). The discrepancies with our data can be related to the different models investigated (rat cell lines versus human primary cultures) as well as to the different TSH concentrations used (around 3 mU/ml in [25], [26], 1 mU/ml in [27], and 0.3 mU/ml in our experiments). The supra-physiological concentrations can induce a higher proliferation rate, with a higher impact on miRNA expression. It is also interesting to note that no common miRNA were detected in [25]–[27].

Beyond treated primary cultures of thyrocytes, we have also investigated thyroid tumors derived cell lines which are an easier and more generally used model to study the molecular features of cancer cells. A previous study of our group [11] has shown that, on the basis of their overall mRNA expression, the different cell lines investigated have evolved into a common dedifferentiated phenotype, even if they retained certain characteristics of the original cancers (such as the causal oncogenic event). This study has also demonstrated that the cell lines have lost the expression of important thyroid specific genes such as TSHR, TPO, Tg or NIS, but not the determination transcription factors as PAX8, FOXE1 (TTF2), NKX2.1 (TTF1). In our study, the question was to see if similar conclusions could be drawn with regard to miRNA expression in those models. To answer this question, six cell lines (TPC1, K1, 8505C, BCPAP, WRO and FTC-133) were analyzed by miRNA microarray and the miRNA expression profiles were compared to those of 8 PTC, 8 FTC and 11 ATC. Analysis of the microarray data has shown clearly that all the cell lines are closer to each other than to their tumors of origin, and are grouped altogether in the same branch of the cluster. Very strikingly, this cluster also included the dedifferentiated and rapidly growing ATC.

Among the 6 cell lines, 19 miRNA were commonly regulated more than 1.5 fold. The comparison of the regulations observed in the cell lines and in the corresponding tumor tissues clearly indicates the similarity of miRNA regulation between these cell lines and ATC, whereas they clearly diverge from the differentiated PTC and FTC from which they were initially derived. This corroborates our previous conclusions suggesting that the cells composing the cell lines have evolved into a common, fully dedifferentiated phenotype and have lost many of their original characteristics. All our 19 miRNA are downregulated in the cell lines and in ATC, suggesting that they may behave as tumor suppressor genes. Some of these miRNA had already been described as regulated in a small ATC cohort by Visone et al. [24] and Braun et al. [23], supporting our results. It is more difficult to compare these results with published data on PTC. Indeed in 10 datasets analysed by Pallante [28], among 37 reported regulated miRNA, only 9 were common to 2 different datasets. Nevertheless 5 of our 19 downregulated miRNA in the cancer cell lines and ATC were reported as downregulated in at least one of these studies: let-7f, miR-26a, miR-138 and miR-141.

Among the commonly regulated miRNA in the 6 studied cell lines, miRNA belonging to 4 families are found (members of miR-135, miR-200, miR-30 and let-7 family). Interestingly, it has been shown that members of the miR-200 family bind and repress Zeb1 and Zeb2, the transcriptional repressors of E-cadherin and inducers of epithelial mesenchymal transition [29]. The downregulation of different members of this family (hsa-miR-141, 200a and 200c) in the cell lines and in ATC could thus participate in promoting EMT. Those miRNA are also regulated by p53 and their downregulation might be correlated with the inactivation of the p53 pathway [12]. Other miRNA modulated in the 6 cell lines are under the control of an important cell regulator. Indeed, members of the let-7 family (hsa-let-7f and hsa-let-7g) as well as hsa-miR-26a and hsa-miR-29a can be repressed by MYC [30]. All these putative tumor suppressor miRNA are downregulated in the 6 cell lines investigated, and their decreased expression may thus have important consequences on the physiology of those cells, namely by contributing to cell proliferation, migration and invasion. Moreover, the pathways targeted by these miRNA have been involved in the pathogenesis of aggressive tumors including ATC. Thus for thyroid cancer cell lines and ATC, the pattern of mRNA expression and the cell phenotypes can be, at least in part, explained by the variations in miRNA expression. The large overlap of miRNA in the totally dedifferentiated cell lines and ATC also fits well with the concept of a fine long-term regulation role of miRNA.

With regard to primary cultures, considering the high number of mRNA that are deregulated after 1.5, 3, 16, 24 and 48 hours of TSH or EGF/serum treatment [8], [9] by opposition to the small number of modulated miRNA, our results suggest that most of the mRNA regulations reported previously [8], [9] are not mediated by miRNA. This also fits well with other characteristics of miRNA regulation, i.e. with the multiplicity of mRNA targets for each miRNA and the multiplicity of miRNA regulating each mRNA. It is also consistent with a role of miRNA as long-term fine tuners, “attenuators” rather than switches of gene expression and in line with the known slow turnover of miRNA, poorly compatible with rapid cell biology regulation [31].

## Supporting Information

Figure S1 **Measurement by qRT-PCR of NIS mRNA expression in primary cultures following TSH or EGF/serum treatment compared to non-treated cells confirming the response of the different primary cultures to the TSH or EGF/serum treatment.** The results are given in log 2 ratio of treated/control mRNA expression. Measurements were done after 48 or 72 h of treatment (PC =  Primary Culture).
(PPTX)

Figure S2 **Validation of the miRNA microarray data.** The expression of 3 down-regulated and one non-regulated miRNA were investigated by qRT-PCR (Taqman) in the 6 cell lines studied by microarray and was compared to a pool of primary cultures (PC) maintained in control medium. U6 SnRNA was used for normalization. Error bars represent the standard deviations.
(PPTX)
