# Supplementary figures and images for: MiRNA Expression May Account for Chronic but Not for Acute Regulation of mRNA Expression in Human Thyroid Tumor Models
Source: PLoS One. 2014 Nov 6;9(11):e111581. doi: 10.1371/journal.pone.0111581 (PMC4222942; doi:10.1371/journal.pone.0111581)

## Slide 1
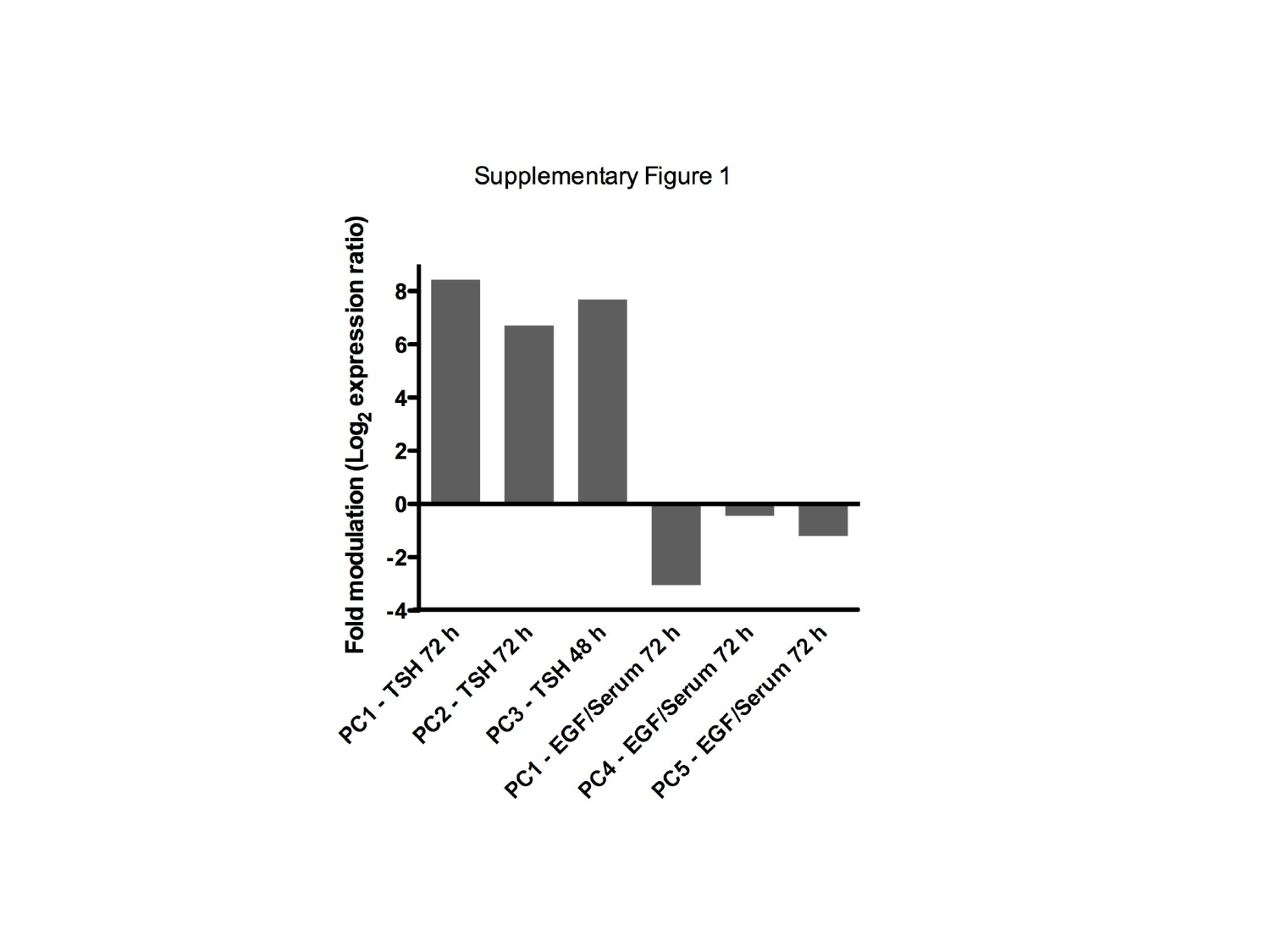

Supplement: Figure S1 — Measurement by qRT-PCR of NIS mRNA expression in primary cultures following TSH or EGF/serum treatment compared to non-treated cells confirming the response of the different primary cultures to the TSH or EGF/serum treatment. The results are given in log 2 ratio of treated/control mRNA expression. Measurements were done after 48 or 72 h of treatment (PC = Primary Culture). (PPTX) [file pone.0111581.s001.pptx]

## Slide 1
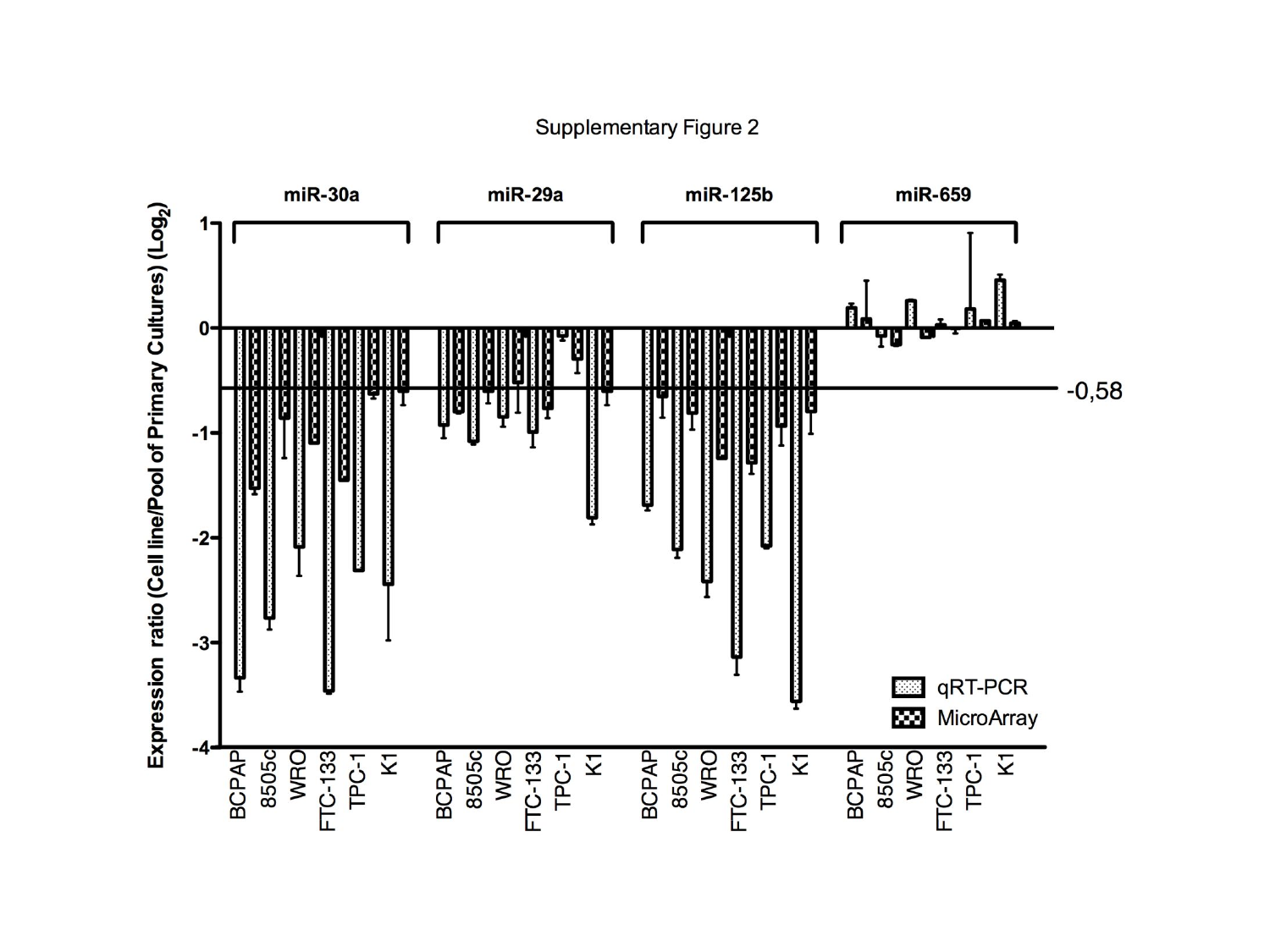

Supplement: Figure S2 — Validation of the miRNA microarray data. The expression of 3 down-regulated and one non-regulated miRNA were investigated by qRT-PCR (Taqman) in the 6 cell lines studied by microarray and was compared to a pool of primary cultures (PC) maintained in control medium. U6 SnRNA was used for normalization. Error bars represent the standard deviations. (PPTX) [file pone.0111581.s002.pptx]
